# Intelligence

**Published:** 1828-06

**Authors:** 


### INTELLIGENCE.

### MONTHLY REPORT OF PREVALENT DISEASES.

The severe north-east winds, which have prevailed during the greater part of the last month, accompanied as they were for many days by the attractive brilliancy of the sun, have produced a decided increase in the list of diseases. Bronchitis has been very common, and, in several cases we have witnessed, more than usually severe. When the complaint had passed on to the chronic state, emetics were of much advantage. Many cases of Paralysis have occurred. Four individuals have been attacked with hemiplegia, in the highest rank of society. In one, electricity was productive of some advantage. During its application, the arm, which at other times was perfectly motionless, was thrown into slight action. In children, remittent fever has been common, and, as usual, tedious in its cure. Ague has also been more than usually frequent for London. We have not met with any case which has resisted the sulphate of quinine, given in one and two grain doses every four or five hours during the intermissions.

---

*Stammering.*—The following certificate has been sent to us for publication, and the respectability of the names attached to it induce us to comply with the request:

The undersigned deem it a duty they owe, not less to the public than to the individuals concerned, to state that on Wednesday morning, the 7th instant, they had an opportunity of witnessing, in the person of a young gentleman,

Mr. H., of Salisbury, a case of as inveterate stammering (especially in reading,) as has ever fallen under their observation. The impediment was not only distressing both to the sufferer and hearer, but was so extreme that scarcely a syllable he undertook to read was rendered intelligible.

The same young gentleman, after having been three days only under the care and treatment of Mr. CLARKE and Dr. ROY, of 41, Park-street, Grosvenor-square, came before us again on Saturday last, when, after an ample investigation, we found him completely cured; and not a trace of his previous malady could be discerned, either in reading or speaking.

*London; May 12th,* 1828.

GRANVILLE SHARP PATTISON,
JAMES JOHNSON, M.D. *Physician to his Royal Highness the Duke of Clarence,*
B. C. BRODIE,
BRANSBY B. COOPER, *Surgeon of Guy's Hospital.*

---

*Anatomy.*—A COMMITTEE of the House of Commons has been for some time engaged in investigating the present difficulties attending the study of anatomy. Among the various speeches made on the occasion, that of Sir JAMES MACKINTOSH was the most interesting, and we are rather surprised that it has not been noticed in any of the periodicals. We subjoin some parts of it, taken from the *Mirror of Parliament.*

That a knowledge of anatomy is to be acquired only by dissection,—that anatomical knowledge is the foundation of medical skill, as well as surgical expertness, and therefore the source of all the benefits which the beneficent arts of medicine and surgery have conferred on mankind, are propositions so nearly self-evident, that I should be ashamed to enforce them by argument before any tolerably well-informed assembly. That the laws, as they are now interpreted and administered, I know in England, and I believe in Scotland, throw obstacles in the way of the acquirement of this knowledge, insurmountable by the majority of those who are bound by law and conscience to acquire it, will, I think, appear with the utmost evidence from a comparison of two indisputable facts. The expense of dissection at Edinburgh is, at this moment, twenty times greater than it is at Paris. The annual number of those who study medicine at the various schools in the Island of Great Britain may be moderately estimated at 2000. It is much if one-fourth of that number be able to bear the expense of foreign residence, and of that preparatory instruction, without which study abroad is impracticable; to say nothing of the time required for such instruction, which would be better employed by the majority on their own profession; or of the weighty consideration that, if anatomy be banished, other medical sciences, which can only be well taught in her neighbourhood, must follow in her train. If, then, the law, and its present administration, go on to produce the full extent of their natural consequences, it is perfectly evident that the remaining three-fourths, by the expense of pursuing anatomy at home, and the expense and loss of time of pursuing it abroad, will be reduced to a state of anatomical ignorance, and consequent professional unskilfulness, of which no man can know that he may not himself be the victim, but by which it may be affirmed, with the most perfect assurance, that the poorer classes must be the chief sufferers, both because they are the poorer, and because they are the most numerous. I entreat the House to ponder this most weighty consideration in their minds. The medical attendants of the superior classes will always be remunerated sufficiently to defray the expenses of a professional education, however they may be enhanced. But the middle classes will, in some degree, suffer; and the poor —that is, the majority and the more helpless portion of the British people,— will, in no long time, be delivered as victims to a race of low and ignorant practitioners. On which side of the question does humanity take her stand? Does she pronounce for the destruction or the protection of anatomy? I desire only that the matter before us may be tried by that issue. I would improve no part of knowledge at the expense of humanity: science is made for man, not man for science. The poorer classes are so much the most numerous, that their well-being must always be coincident with that of mankind. I earnestly beseech the House to consider their deep stake in this question. I feel a strong assurance that the growing intelligence of the people will resist every attempt to rouse the passions to arms against their own healths and lives. They have already outgrown prejudices as natural and equally mischievous. No incendiary can any longer lead them to destroy a granary. They show admirable sense, even in the midst of suffering, on the perplexing subject of machinery. They will, I trust, soon learn that the dissecting-room also is more essential to their welfare than to that of any other part of society.

Allow me to illustrate, by an imaginary case, the opposite effects of continuing to bar up, and of trying to widen, the only access to anatomical knowledge. If we were told that, in some desert region of central Africa, it was the practice of a tribe of savages to put to death annually a certain number of their own sick and wounded, we should surely listen to the story with a hope to find it false. But, if it were added that these murders were perpetrated, not by the instantaneous and merciful operation of the sword, the pistol, or the axe, but by a lingering torture for months or years, we would require the strongest evidence to induce us even to listen to such a charge against cannibals themselves. If we are told that we are ourselves chargeable with equal barbarity, should we not cry out, with the Syrian of old, " Is thy servant a dog, that he should do this thing?" But let us look at home. Let us not suffer ourselves to be paid in words, which, as was sharply and sagely said, " are the counters of wise men and the money of fools." What is the substantial difference between the supposed barbarity in Africa and the legal impediments to anatomy in Great Britain? In proportion to every degree in which anatomy declines and medicine sinks, an additional number of human lives must be cut short. If the healing arts preserve life, their decay must destroy it. That their improvement has contributed to that prolongation of its average duration which has taken place during the last half century, is what nobody but the most extravagant dealers in common-place paradox will venture to dispute. The mean length of life chiefly depends on the treatment of children, and the decline of medical science must be attended, in its very beginning by a real, though not nominal, massacre of infants. If, indeed, it were to kill at a blow, it might be a blessing to many. But its victims will " die so slowly, none can call it murder." The bungling surgeon will make his instrument a means of more cruelty than the tomahawk; the ignorant physician will kill only by the protracted torture of disease.

Let every man, who calls out law or prejudice against dissection, consider whether he does not do his utmost to abate the means of lengthening life and (what is more important) of alleviating misery. Let him deeply reflect, whether an inconsiderate word may not make an orphan, and an inflammatory sentence may not cause unspeakable agony to hundreds. What a fearful responsibility does he incur to all those who may suffer from the blow which he has struck against the healing arts!

I should be most painfully perplexed if I thought myself, in this case, reduced to the sad necessity of choosing between the means of relief to bodily suffering, and the discipline which cultivates our moral feelings. I am not among those who underrate the rites of sepulture; still less the regard for the remains of the dead, which has prompted mankind in every age to hold these rites sacred. I believe that such a regard is inseparable from affection towards the living. As the cannibal feeds his ferocity by vindictively devouring the flesh of his enemies, so, it seems to me, funeral honours may be said, in some measure, to return and replenish those sacred fountains of kindness and compassion from which they flow. But I will not believe that the moral culture of man is at variance with his bodily welfare. I am convinced that inquiry will discover means, sanctioned by the experience of other countries, by which, while the noble science of anatomy, and the beneficent arts of medicine and surgery, are preserved among us, the alarms of affection may be appeased, and the sanctuary of the grave rendered more inviolable. I believe that a plan may be found, which will spare the feelings of every known or discoverable person; and I conceive that, to require more, would be fantastic extravagance. I believe, with equal confidence, that, if things go on as they now threaten, we shall close the better part of the means of instruction in the medical sciences; but that a miserable remnant must still be scantily supplied by that system of clandestine and contraband disinterment, which shocks the heart of the mourner,—degrades science, as well as renders its profession odious; and becomes, like smuggling and poaching, a school in which men are fitted for the worst crimes.

As to the dissection of suicides, which has been alluded to, it would surely be an act of wanton cruelty to survivors in all cases of insanity, and indeed in most others. London is the capital city in Europe where they are the most rare. The number is considerably greater in Paris, where the population is about one half. The dissection of murderers is evidently a resource of no importance. The average number of executions for murder in England and Wales has, during the last seven years, been only fourteen, or one in 850,000 persons. In London, the average of the same executions in the same time has been only five, being something more than one in 300,000 persons. When dissection was made part of the punishment for murder, it was a much more considerable resource to anatomy, which then demanded much less. According to a calculation which I have made with some care, it appears that nearly a century ago, in the year 1733, the whole executions in London were to the population as one to 10,000. In the last seven years, the whole executions in London have been to the population as one to 70,000. The relative number of executions for murder in the capital has diminished in a much greater proportion than that for all offences. I believe that the dissections for murder are injurious to the anatomist, by bringing new unpopularity upon his most beneficial occupation. It is at least certain that they are useless.

Positively to add to the terrors of the punishment of death, is impracticable in a civilised age. There is only one way in which penal law can contribute to keep up the national abhorrence of murder. It is by confining the punishment of death to that crime, and to those acts of cruelty, violence, or destruction, which partake of its nature, and often lead to its perpetration. The laws which alike punish with death the murderer of his father, the stealer of a sheep, and the forger of a bank-note, do their worst to drag down parricide to the level of sheep-stealing. It is no objection to this truth, that the humanity of Englishmen, formed and fostered by far other institutions, has hitherto maintained a successful struggle against these barbarising laws. We cannot add to capital punishment, but we may mightily strengthen its power by bringing its whole force to bear on the most atrocious crimes.

---

*Bill to regulate the care of Insane Persons.*—A BILL is now in progress through Parliament for the above purpose, and some idea may be gathered of its nature and tendency from the following observations, which we condensed from an anonymous pamphlet, containing some sensible remarks on the subject:

A party applying for a license is required, in the first place, to send in a most minute plan of the house, with a description of every room and apartment therein; and a statement of the number of patients to be received, and of keepers, distinguishing male from female, and servants to be employed therein; and if any addition or alteration shall be made to the house, notice is to be given, and an amended plan to be sent in within seven days.

In large establishments, a compliance with these regulations will, at all times, be extremely expensive and troublesome, and in many instances, owing to the great fluctuation in the number of patients, almost impracticable. Besides, the number varies with the rank and fortune of patients. One patient sometimes occupies several apartments; sometimes several inferior patients occupy a single room: either class may preponderate. Neither can it be known beforehand how many patients, or how many of either sex, there will be in a house at any one time; and, as the number of keepers and servants is regulated both by the rank as well as by the number and sex of the patients, it is impossible to state how many attendants will be kept. From the same causes, frequent alterations in the internal arrangements of the house become necessary. Yet, in all such cases, new plans are to be verified by the personal inspection of the clerk to the commissioners.

The license, too, is to be paid for according to the number of patients to be received: a regulation which, for the above reasons, cannot be strictly complied with; and, where two or three patients only are kept, £15 is too heavy a charge. £10 was before paid for a license for less than ten patients.

The notice of application for a license, and the license itself, is to contain the name and place of abode, not only of the person to whom the same is to be granted, but of every person interested in the profits of the house.

This is a useless inquisition into private concerns, as the person to whom the license is granted is alone responsible. It will likewise render it next to impossible to obtain pecuniary assistance towards placing an establishment on a respectable and proper footing; because advances for such purposes must, in most instances, be secured on the profits, and many people will object to having their names or property exposed to such hazardous contingencies as all these establishments would be rendered liable to.

The commissioners for the London district, and the justices of the peace in counties, may refuse to grant or renew the annual license, ***without assigning any reason;*** and against such decision there is no appeal!

Licenses may be revoked also by the same authorities; and such refusal or revocation is forthwith to be published in the London Gazette, or a county newspaper. If, after such publication, the proprietor continues to keep two or more patients for fourteen days, he is declared guilty of a misdemeanour. No provision, however, is made for the disposal of the patients in such event. In many instances their friends reside on the continent, or in the East or West Indies; or there is no immediate relation who will receive them, or they are wards of the Court of Chancery; and in that time, perhaps, the patient could not be disposed of. The proprietor of the house must either turn all these helpless beings adrift, or retain them at the risk of a prosecution.

In houses situate out of the London district, the number of visitors is unlimited.

The commissioners are authorised to visit houses by night as well as by day. The exercise of this power will always be attended with the most injurious consequences to patients, independent of the stigma thereby necessarily cast on the superintendent of the house. Night visits could not be made without disturbing and violently agitating most of the inmates. To delicate females especially, as well as to convalescents, whether actually inspected or not, such a visitation would probably prove dangerous.

The lord chancellor, and either of the lords chief justices, may direct, not merely, as at present, the commissioners alone, but any person whatever, to visit any house; and a similar power is now given to the secretary of state for the home department.

In addition to the regular commissioners and visitors, the rector, vicar, or curate of the parish, is likewise authorised, with their consent, to visit houses, though no adequate reason can well be assigned why such visitation should be permitted; and, as the superintendent is to admit or refuse the clergyman at his own discretion, such a clause is perfectly nugatory, but yet may raise much ill-will between the resident clergyman, the visitors, and the superintendent.

The *lunatics* themselves, too, are at liberty to require the assistance of any minister or spiritual teacher; and the reason for refusing it is to be registered! The patient, however, is the last person to whom such a power ought to be given; but, when he learns that the law sanctions his request, it will be constantly preferred, and the refusal of it be a cause of prejudice against the superintendent, for whom every patient ought to possess great respect.

In cases where the disease is connected with religious feelings, such communications, if indulged, might tend to increase it; and a refusal, though in itself judicious and necessary, will almost invariably cause irritation in the mind of the patient.

The certificate necessary to the admission of a patient is to be signed by two medical practitioners, who shall have *separately* examined the party.

The commissioners and visitors are empowered to discharge patients; yet, in many instances, they must, particularly if not medical men, be wholly incompetent to decide on the propriety of such discharge. A more striking example of this cannot be given than is to be found in the evidence of Dr. Latham, the president of the College of Physicians, before the committee of the House of Commons, in 1815, which shall be stated in his own words:

"The first time I was a commissioner, we examined a house at Plaistow: there were two women confined, whom I thought were not insane. The keeper said they were, and that we were mistaken. We desired them to write to their friends to give them a trial. We were all of opinion these women were improperly confined, and desired their friends would take them out. Upon our next visitation, the following year, I had, of course, considerable curiosity to know what had become of these two people: one had drowned herself, and the other had hanged herself."

The clauses authorising one commissioner or justice of the peace to order the secretary of the commissioners, or clerk of the peace, to divulge to any person the name and place of residence of any lunatic; requiring a return to be made of a single patient received into a house; and empowering the lord chancellor, or secretary of state, to appoint any person to visit any lunatic under the care of a relation or friend, are destructive of all that privacy which is so truly desirable for the patient, and a most serious inquisition into domestic afflictions. Misfortunes, which every relation is anxious to keep secret, on account of the patient as well as of himself, will thus be made public; so that the very object which the legislature professes to have in view—that of ameliorating the condition of the unhappy persons subject to this malady, will be, in respect to those insane who demand the most delicate consideration, entirely defeated.

How far the power given to *visit and examine any insane person who shall be confined, in the care of any relative or friend, or in the exclusive care of any other person,* is or is not an outrage on domestic privacy, and the free agency which every Englishman has felt himself hitherto entitled to exercise in his own family, every one is able to judge; but those can best appreciate who are personally interested in this extraordinary enactment.

---

We understand that Dr. Birkbeck is about retiring from the appointment of Physician to the General Dispensary, Aldersgate-street. The present candidates for the office are Dr. Woodforde and Dr. C. J. Roberts.

### METEOROLOGICAL JOURNAL,

*From April 20th, to May 20th, 1828.*

By Messrs. Harris and Co. Mathematical Instrument Makers, 50, High Holborn.

| April | Rain gauge. | Moon. | Thermom. | | | Barometer. | | De Luc's Hygrom. | | Winds. | | Atmospheric Varaitions. | | |
|---|---|---|---|---|---|---|---|---|---|---|---|---|---|---|
| | | | 9 A.M. | MAX. | MIN. | 9 A.M. | 10 P.M. | 9 A.M. | 10 P.M. | 9 A.M. | 10 P.M. | 9 *a.m.* | 2 *p.m.* | 10 *p.m.* |
| 20 | | | 47 | 50 | 43 | 29.67 | 29.57 | 98 | 98 | SE | ENE | Cloudy | Cloudy | Sleet |
| 21 | .15 | | 46 | 49 | 39 | 29 60 | 29.54 | 96 | 98 | NE | N | Rain | Show'ry | Rain |
| 22 | | ☽ | 44 | 50 | 44 | 29.51 | 29.60 | 94 | 98 | W | WSW | Cloudy | Cloudy | Cloudy |
| 23 | . | | 49 | 56 | 47 | 29.67 | 29.70 | 97 | 98 | NW | W | — | — | Rain |
| 24 | | | 55 | 60 | 50 | 29.67 | 29.76 | 86 | 94 | NW | W | Fair | Fair | Fair |
| 25 | | | 56 | 59 | 47 | 29.73 | 29.62 | 92 | 84 | SW | WSW | — | Cloudy | Cloudy |
| 26 | | | 54 | 59 | 46 | 29.85 | 30.01 | 80 | 79 | NW | WNW | — | Fine | Fine |
| 27 | | | 60 | 65 | 48 | 30.06 | 30.10 | 80 | 80 | W | WSW | — | — | — |
| 28 | | | 64 | 70 | 53 | 30.14 | 30.15 | 80 | 78 | SW | SW | — | — | — |
| 29 | | ○ | 68 | 72 | 57 | 30.11 | 30.07 | 75 | 84 | SW | WSW | — | — | — |
| 30 | | | 62 | 64 | 48 | 30.19 | 30.21 | 90 | 90 | NNE | SE | — | — | — |
| May | | | | | | | | | | | | | | |
| 1 | | | 54 | 63 | 45 | 30.24 | 30.18 | 90 | 90 | S | ENE | — | — | Fair |
| 2 | | | 51 | 61 | 48 | 30.12 | 30.03 | 90 | 94 | SE | SE | — | — | Cloudy |
| 3 | | | 52 | 60 | 50 | 29.98 | 29.84 | 96 | 98 | SE | S | Rain | Rain | — |
| 4 | .13 | | 58 | 63 | 48 | 29.76 | 29.63 | 98 | 98 | WNW | NNW | — | — | Fair |
| 5 | | | 57 | 59 | 46 | 29.57 | 29.57 | 85 | 86 | NW | NW | Cloudy | Fair | — |
| 6 | | ☾ | 54 | 58 | 45 | 29.60 | 29.67 | 88 | 92 | W | NNW | Fair | — | — |
| 7 | | | 51 | 60 | 46 | 29.71 | 29.75 | 97 | 98 | NE | NE | — | — | Rain |
| 8 | | | 47 | 59 | 43 | 29.75 | 29.83 | 98 | 96 | E | ENE | Rain | — | Fine |
| 9 | | | 54 | 62 | 47 | 29.92 | 30.02 | 90 | 88 | N | WNW | Fair | Fine | — |
| 10 | | | 57 | 66 | 53 | 30.04 | 30.00 | 80 | 90 | WNW | W | — | — | — |
| 11 | | | 59 | 63 | 51 | 30.00 | 30.04 | 85 | 88 | ENE | ENE | — | — | — |
| 12 | | | 54 | 64 | 47 | 30.07 | 30.16 | 88 | 87 | ENE | ENE | — | — | — |
| 13 | | ● | 57 | 65 | 51 | 30.12 | 30.09 | 85 | 92 | SW | ENE | — | — | — |
| 14 | | | 62 | 65 | 47 | 30.05 | 30.00 | 90 | 90 | E | E | — | — | — |
| 15 | | | 58 | 67 | 58 | 29.94 | 29.85 | 90 | 90 | ESE | E | — | — | — |
| 16 | | | 63 | 70 | 57 | 29.74 | 29.74 | 90 | 90 | E | ENE | — | — | — |
| 17 | | | 64 | 68 | 56 | 29.70 | 29.69 | 90 | 94 | E | E | — | — | Sleet |
| 18 | | | 58 | 63 | 47 | 29.70 | 29.70 | 92 | 89 | SSE | ESE | — | — | Fine |
| 19 | | | 58 | 63 | 48 | 29.70 | 29.71 | 88 | 78 | SSE | E | — | — | — |

The quantity of Rain fallen in the month of April, was 1 inch and 95.100ths.

